# Supplementary material for: A standardized gnotobiotic mouse model harboring a minimal 15-member mouse gut microbiota recapitulates SOPF/SPF phenotypes
Source: Nat Commun. 2021 Nov 18;12:6686. doi: 10.1038/s41467-021-26963-9 (PMC8602333; doi:10.1038/s41467-021-26963-9)
Supplement: Supplementary file 1 — Supplementary Information [file 41467_2021_26963_MOESM1_ESM.pdf]

Supplementary Information

A standardized gnotobiotic mouse model harboring a minimal 15-member mouse gut microbiota recapitulates SOPF/SPF phenotypes

Marion Darnaud[1]*, Filipe De Vadder[2], Pascaline Bogeat[1], Lilia Boucinha[1], Anne-Laure Bulteau[2], Andrei Bunescu[1], Céline Couturier[1], Ana Delgado[1], Hélène Dugua[1], Céline Elie[1], Alban Mathieu[1], Tereza Novotná[3], Djomangan Adama Ouattara[1], Séverine Planel[1], Adrien Saliou[1], Dagmar Šrůtková[3], Jennifer Yansouni[1], Bärbel Stecher[4,5], Martin Schwarzer[3§], François Leulier[1,2§], Andrea Tamellini[1§]

[1] BIOASTER, Institut de Recherche Technologique, 40 avenue Tony Garnier, 69007 Lyon, France.

[2] Institut de Génomique Fonctionnelle de Lyon, Université de Lyon, Ecole Normale Supérieure de Lyon, Centre National de la Recherche Scientifique, Université Claude Bernard Lyon 1, Unité Mixte de Recherche 5242, 46 Allée d'Italie, 69364 Lyon, Cedex 07, France.

[3] Laboratory of Gnotobiology, Institute of Microbiology of the Czech Academy of Sciences, 54922 Nový Hrádek, Czech Republic.

[4] Max von Pettenkofer Institute of Hygiene and Medical Microbiology, Ludwig-Maximilians-University of Munich, 80336 Munich, Germany.

[5] German Center for Infection Research (DZIF); Partner Site Munich.

§Co-Senior authorship

*Corresponding author: gnotobiology@bioaster.org

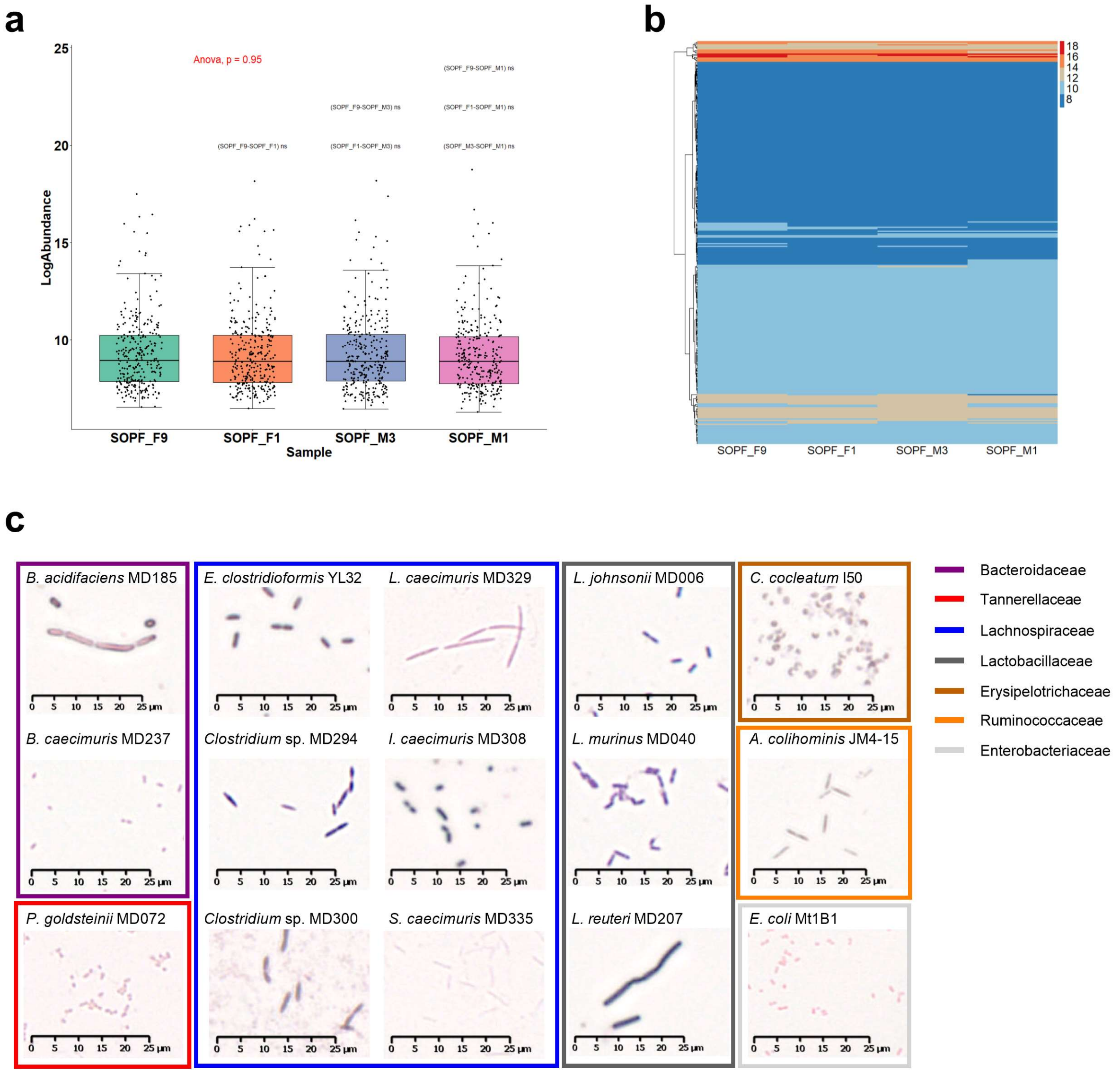

Supplementary Fig. 1. Distribution of bacterial families among the C57BL/6J SOPF mice used to design the GM15 community and morphological features of the GM15 strains. a Box plots extend from the 25th to 75th percentiles and show centre line as median. Whiskers represent lower and upper value within the 1.5 fold of the interquartile range, and dots represent individual bacterial families (n=296). ANOVA and two-tailed paired t-test. **b** Heatmap representing the distribution and classification of the log-normalized abundance of each bacterial family (lines, n=296) for each mouse (columns, n=4). **c** Representative images out of three independent experiments performed by different operators. Each bacterial strain was grown individually from a single colony isolated on agar medium and amplified in liquid culture to exponential growth phase. Bacteria were Gram-stained and imaged by light microscopy (80-

fold magnification, NanoZoomer S60, Hamamatsu). Members of *Bacteroidaceae*, *Tannerellaceae* and *Enterobacteriaceae* stained Gram-negative, while *Lactobacillaceae*, *Erysipelotrichaceae* and *Ruminococcaceae* stained Gram-positive. *Lachnospiraceae* stained Gram-positive, except *Longibacillum caecimuris* MD329 and *Subtilibacillum caecimuris* MD335, which stained Gram-negative likely due to their cell wall structure as already reported for other Clostridiales [1]. Source data are provided as a Source Data file.

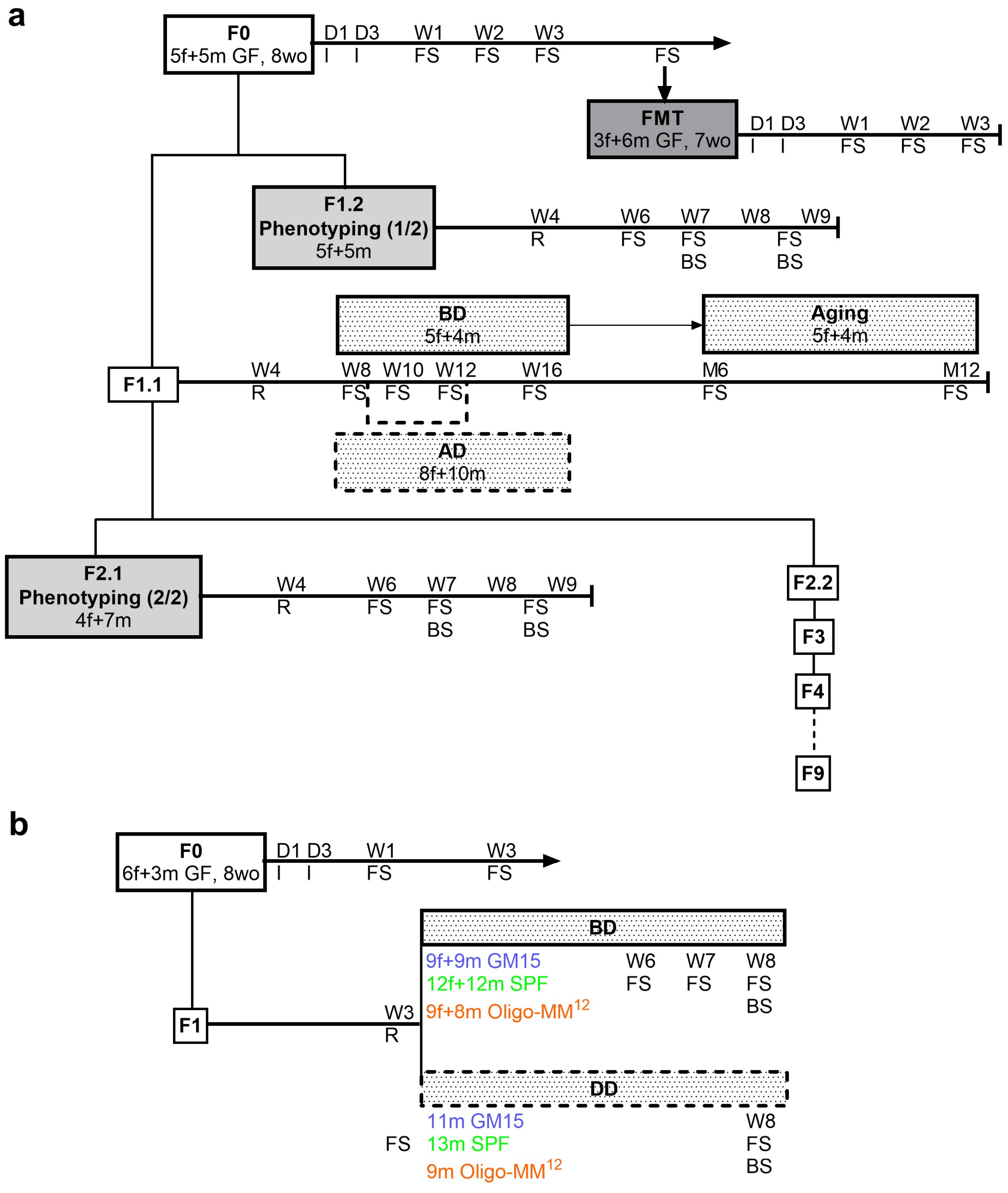

AD: alternative diet; BD: breeding diet; BS: blood sampling; D: day; DD: depleted diet; f: female; F: filial generation; FMT: fecal microbiota transplantation; FS: fecal sampling; GF: germ-free; I: inoculation of bacteria; m: male; M: month; R: randomisation at weaning; SPF: specific pathogen-free; W: week; wo: weeks old

Supplementary Fig. 2. *In vivo* experimental design. a Facility 1. First, 5 couples of 8 week-old C57BL/6J GF mice were colonized by oral gavage with the fresh frozen GM15 bacterial community, twice at 48h interval (F0). GM15 microbiota stability was assessed by qPCR microfluidic assay from feces collected after 1, 2 and 3 weeks. Then, in order to evaluate the

reproducible transfer of the GM15 microbiota by fecal microbiota transplantation, 7 week-old C57BL/6J GF mice were orally gavaged with a suspension of fresh fecal pellets from GM15 mice (F0), twice at 48h interval. Again, qPCR microfluidic assay from feces collected after 1, 2 and 3 weeks was carried out. Next, the GM15 mouse line was amplified to monitor the GM15 microbiota through nine filial generations at 6 weeks of age (F1-F9), and allow the phenotyping study from two consecutive generations (F1.2 and F2.1). Reproduction performance and perinatal mortality were recorded, 4 week-old mice were randomly selected at weaning, monitored weekly for body weight and size, and feed intake, until sacrifice at 8-9 weeks of age. GF and SOPF mice were also studied as control groups. Besides, a comparative analysis was done on the fecal microbiota of 8 week-old GM15 mice (F1.1) either fed with the breeding diet or an alternative isocaloric diet given for 4 weeks. Finally, the fecal microbiota of 6 month-old and 12 month-old control GM15 mice fed with the breeding diet was analyzed. **b** Facility 2. First, 3 trios of 8 week-old C57BL/6J GF mice were colonized by oral gavage with the fresh frozen GM15 bacterial community, twice at 48h interval (F0). GM15 microbiota stability was assessed by qPCR microfluidic assay from feces collected after 1 and 3 weeks. Next, the GM15 mouse colony was amplified to monitor the GM15 microbiota in generation F1 at 6 weeks of age, to allow the phenotyping study and to assess mice’ response to diet-induced stunting.

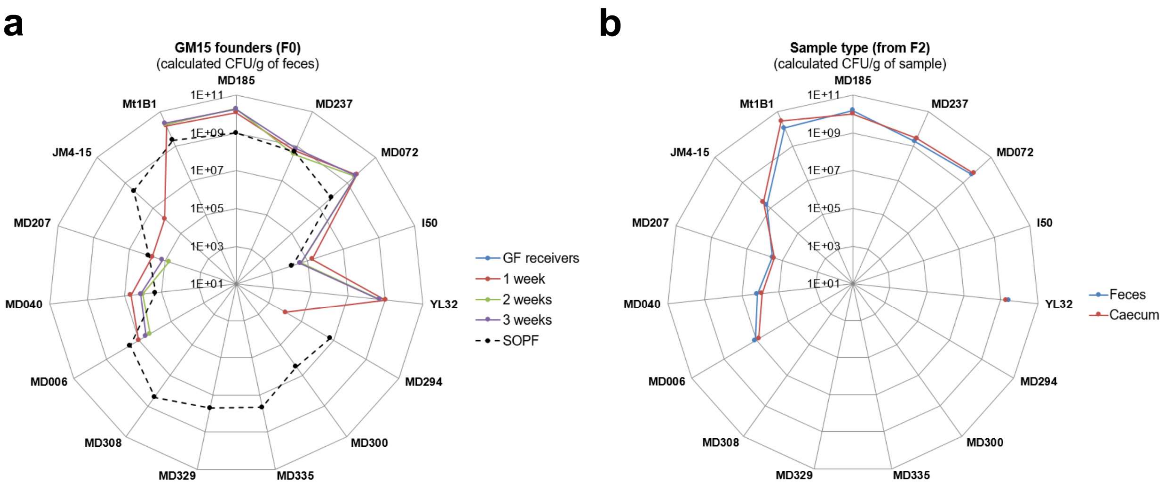

Bacteroides acidifaciens MD185
Bacteroides caecimuris MD237
Parabacteroides goldsteinii MD072
Clostridium cocleatum I50 *
Enterocloster clostridioformis YL32
Clostridium sp. MD294 *
Clostridium sp. MD300 *
Subtilibacillum caecimuris MD335 **
Longibacillum caecimuris MD329 **
Irregularicoccus caecimuris MD308 **
Lactobacillus johnsonii MD006
Ligilactobacillus murinus MD040
Limosilactobacillus reuteri MD207
Anaerotruncus colihominis JM4-15 *
Escherichia coli Mt1B1

Supplementary Fig. 3. Assessment of gut microbiota stability in GM15 founders and of reproducibility between fecal and caecal samples of individual GM15 mice. SOPF group shows the distribution of each GM15 strains in the complex gut microbiota of 8-week-old SOPF mice. The absolute quantification of each strain was determined by specific qPCR microfluidic assay. * Strains I50, MD294, MD300 and JM4-15 were at the detection limit of the qPCR microfluidic assay, and thus were not detected in all samples. ** Strains MD335, MD329 and MD308 were below detection limit of the qPCR microfluidic assay. Strain YL32, obtained from the DSMZ collection, was not detected in our SOPF colony. **a** Radar plot showing the GM15 strains distribution in feces of C57BL/6J GF mice before and 1, 2 and 3 weeks after the oral colonization with the GM15 community (F0, n=10). **b** Radar plot showing the reproducible detection of the GM15 strains in feces and caecum collected from the same GM15 mice (F2, n=11). Source data are provided as a Source Data file.

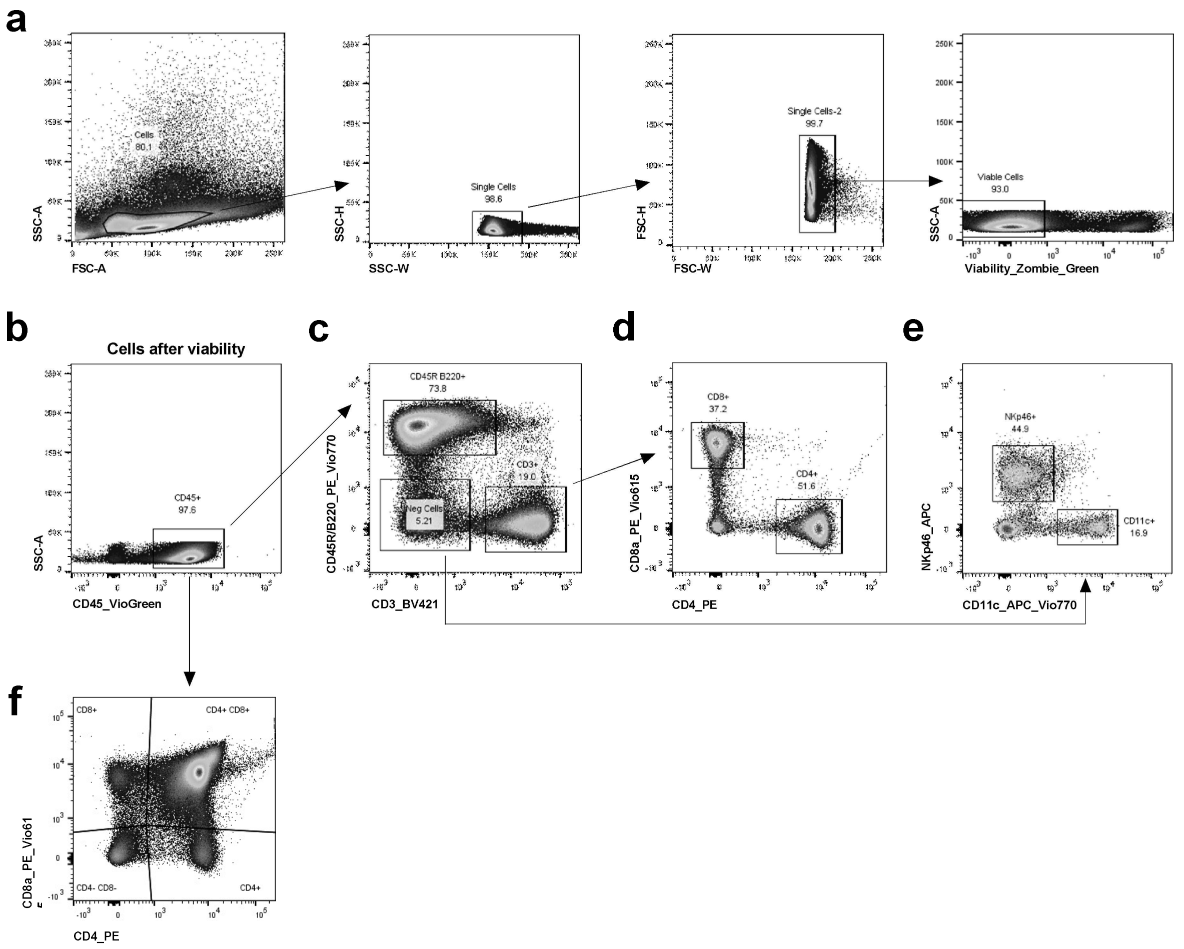

Supplementary Fig. 4. Gating strategies used to define different cell subpopulations in whole blood, mesenteric lymph nodes, Peyer's patches, spleen and thymus from GF, GM15 and SOPF mice for immunophenotyping. a Gating strategy to analyze viable cells after exclusion of doublets. A numerical value for percentage of gated cells is presented. **b** Gating strategy to analyze CD45+ cells (Fig. 4f-i). **c** Gating strategy to analyze CD45R/B220+ cells (Supplementary Fig. 5a,c). **d** Gating strategy to analyze CD4+ cells (Supplementary Fig. 5e) and CD8+ cells (Supplementary Fig. 5c), except from thymus. **e** Gating strategy to analyze CD11b+ or CD11c+ cells (Supplementary Fig. 5a,b,e) and NKp46+ cells (Supplementary Fig. 5b,e), except from thymus, which was not analyzed. **f** Gating strategy to analyze CD4+ cells (Supplementary Fig. 5d) and CD8+ cells from thymus.

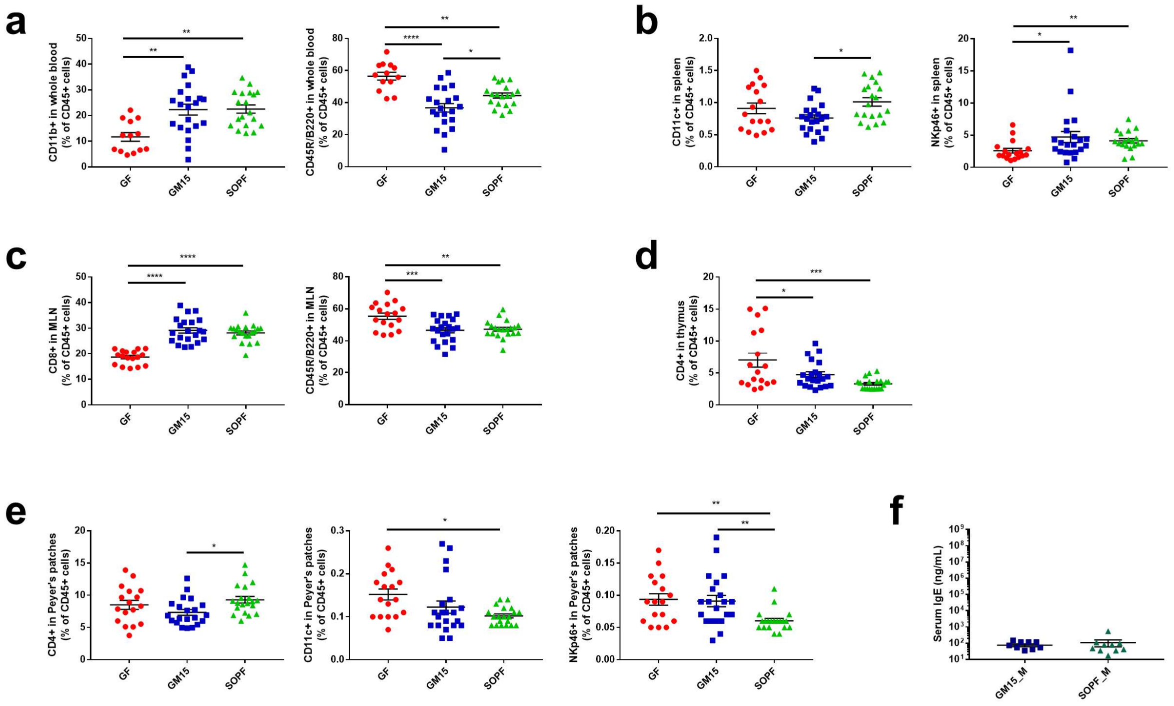

Supplementary Fig. 5. Immune cell populations profiling analysis by flow cytometry in different organs from mice from two consecutive filial generations (F1-F2) and IgE titration by ELISA in additional mice (F5-F6). a-e Dot plots where dots, lines and error bars represent respectively individual mice, means and SEM. Data are represented as % of CD45+ cells. **a** Monocytes and B cells population in whole blood. One-way ANOVA followed by Tukey's multiple comparison analyses (13 GF, 21 GM15 and 19 SOPF). **b** DC and HN cells in spleen. One-way ANOVA followed by Tukey's multiple comparison analysis (DC) and Dunn's multiple comparison analysis (NK cells) (17 GF, 21 GM15 and 19 SOPF). **c** CD8+ T cells and B cells in MLN. One-way ANOVA followed by Tukey's multiple comparison analyses (17 GF, 21 GM15 and 20 SOPF). **d** CD4+ T cells in thymus. One-way ANOVA followed by Tukey's multiple comparison analysis (17 GF, 21 GM15 and 20 SOPF). **e** CD4+ T cells, DC and NK cells in PP. One-way ANOVA followed by Tukey's multiple comparison analysis (CD4+ T cells) and Dunn's multiple comparison analyses (DC and NK cells) (17 GF, 21 GM15 and 20 SOPF). **f** Dot plots where dots, lines and error bars represent respectively individual mice, means and SEM. IgE ELISA assay. Two-tailed Mann-Whitney test (additional F5-F6 male mice: 10

GM15_M and 10 SOPF_M). *P<0.05, **P<0.01, ***P<0.001, ****P<0.0001. Source data are provided as a Source Data file.

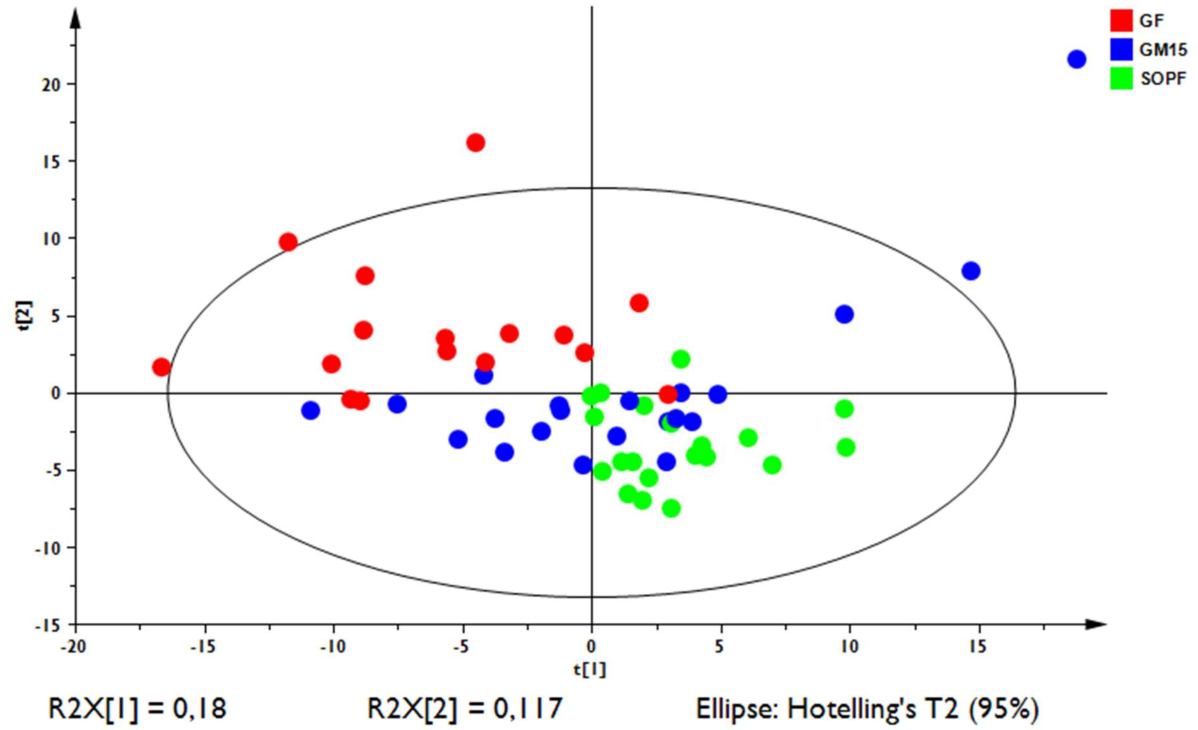

Supplementary Fig. 6. PCA screen plot based on ^{1}H NMR plasma fingerprints. Processed ^{1}H NMR plasma spectra of polar metabolites were binned with AMIX v3.9.14 software from Bruker Biospin, using 0.04 ppm width from 0.5 to 10 ppm spectral window. The residual water region from 4.68 to 4.88 ppm was excluded from analysis. All spectra were normalized to the total spectral area and the data table was exported into SIMCA v13.0.3 software for statistical analysis. The PCA analysis was performed using UV-scaling of data and the model was autofit using cross validation rules to determine the number of significant components. The clouds of sample points GF (n=16), GM15 (n=21) and SOPF (n=20) are distributed according to microbiota complexity from left to right on PC1 (18%) and top to down on PC2 (12%). Source data are provided as a Source Data file.

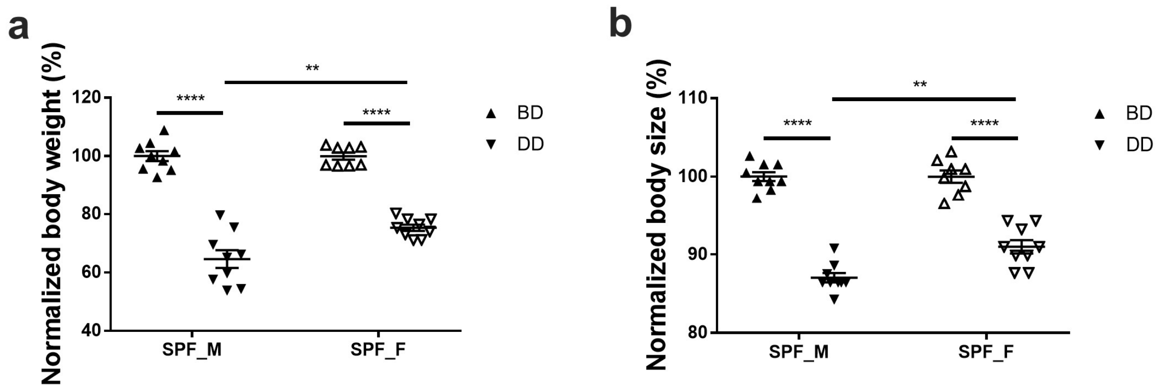

Supplementary Fig. 7. Diet-induced stunting is more prominent in BALB/c male mice compared to female mice. Dot plots where dots, lines and error bars represent respectively individual mice, means and SEM. Specific pathogen-free (SPF), male (M), female (F), breeding diet (BD) and depleted diet (DD) respectively. 9 animals per group, except 8 SPF_F_BD. Mice were fed with either BD or DD between day 21 (at weaning) and day 56 after birth. Normalized body weight (**a**) and size (**b**) as percentages of the average body weights of animals of the same age (56 days old) and same sex fed with BD. Two-tailed Mann-Whitney test was used to analyze normalized body weight data of SPF_F_BD, otherwise two-tailed unpaired t-test was applied. *P<0.05, **P<0.01, ***P<0.001, ****P<0.0001. Source data are provided as a Source Data file.

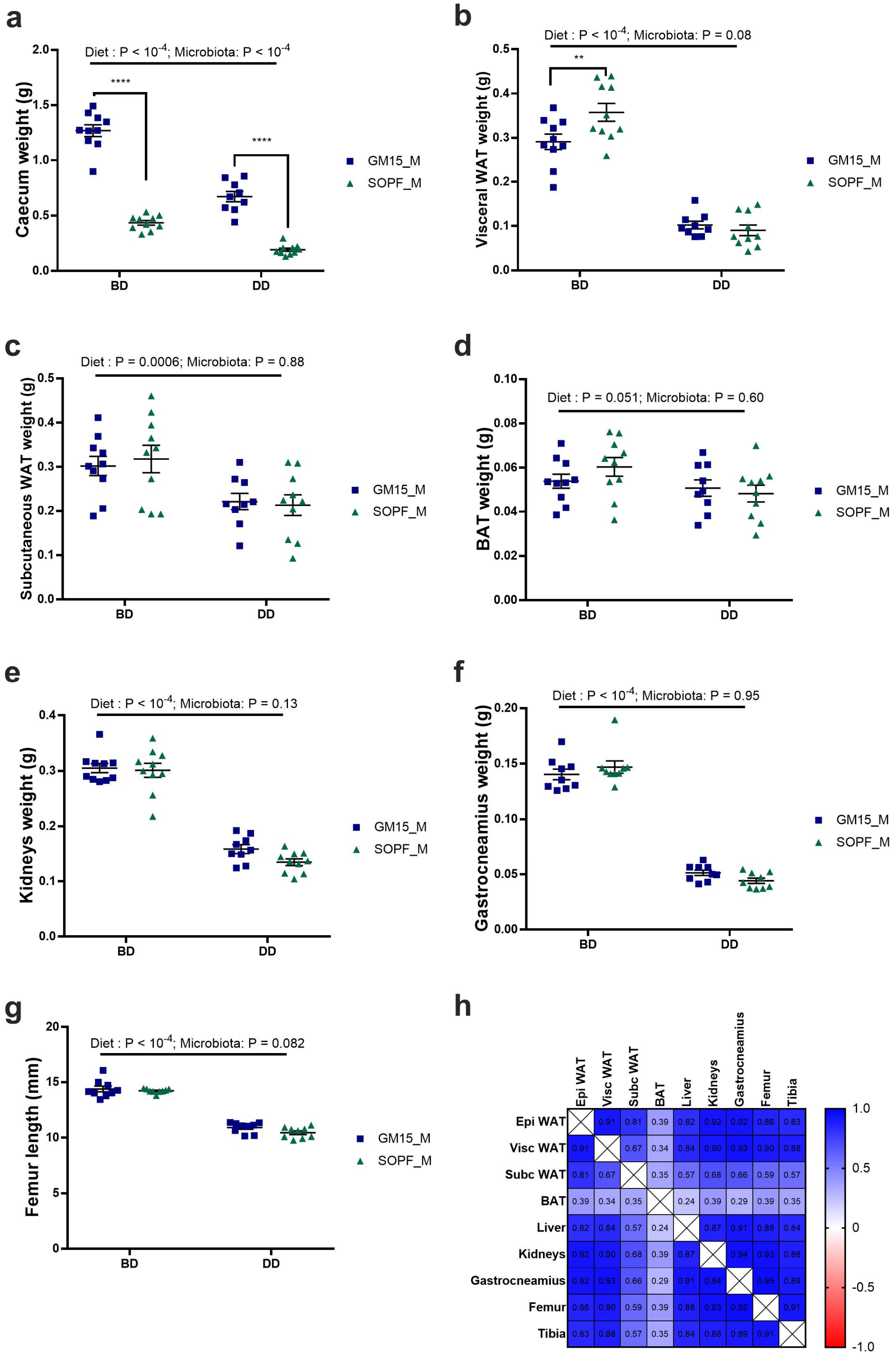
a
Diet : P < 10-4; Microbiota: P < 10-4
****
****
Caecum weight (g)
BD
DD
GM15_M
SOPF_M
b
Diet : P < 10-4; Microbiota: P = 0.08
**
Visceral WAT weight (g)
BD
DD
GM15_M
SOPF_M
c
Diet : P = 0.0006; Microbiota: P = 0.88
Subcutaneous WAT weight (g)
BD
DD
GM15_M
SOPF_M
d
Diet : P = 0.051; Microbiota: P = 0.60
BAT weight (g)
BD
DD
GM15_M
SOPF_M
e
Diet : P < 10-4; Microbiota: P = 0.13
Kidneys weight (g)
BD
DD
GM15_M
SOPF_M
f
Diet : P < 10-4; Microbiota: P = 0.95
Gastrocneamius weight (g)
BD
DD
GM15_M
SOPF_M
g
Diet : P < 10-4; Microbiota: P = 0.082
Femur length (mm)
BD
DD
GM15_M
SOPF_M
h
Epi WAT
Visc WAT
Subc WAT
BAT
Liver
Kidneys
Gastrocneamius
Femur
Tibia
Epi WAT 0.91 0.81 0.39 0.82 0.92 0.92 0.86 0.83
Visc WAT 0.91 0.67 0.34 0.84 0.90 0.93 0.90 0.88
Subc WAT 0.81 0.67 0.35 0.57 0.68 0.66 0.59 0.57
BAT 0.39 0.34 0.35 0.24 0.39 0.29 0.39 0.35
Liver 0.82 0.84 0.57 0.24 0.87 0.91 0.88 0.84
Kidneys 0.92 0.90 0.68 0.39 0.87 0.94 0.93 0.88
Gastrocneamius 0.92 0.93 0.66 0.29 0.91 0.94 0.95 0.89
Femur 0.86 0.90 0.59 0.39 0.88 0.93 0.95 0.91
Tibia 0.83 0.88 0.57 0.35 0.84 0.88 0.89 0.91
1.0
0.5
0
-0.5
-1.0

Supplementary Fig. 8. Measurements of several organs after nutritional challenge. Dot plots where dots, lines and error bars represent respectively individual mice, means and SEM. Male (M), breeding diet (BD) and depleted diet (DD) respectively. Measurements at day 56 of weights of caecum (**a**), visceral (**b**) and subcutaneous white adipose tissue (WAT) (**c**), brown adipose tissue (BAT) (**d**), kidneys (**e**) (10 males per group, except 9 GM15_M_DD), and gastrocneamius muscle (**f**) and length of right femur (**g**) (9 males per group). P-values after two-way ANOVA were adjusted for Sidak's post-hoc test for multiple comparisons. **h.** Correlation matrix showing Pearson r values of all the measured parameters (9 males per group). *P<0.05, **P<0.01, ***P<0.001, ****P<0.0001. Source data are provided as a Source Data file.

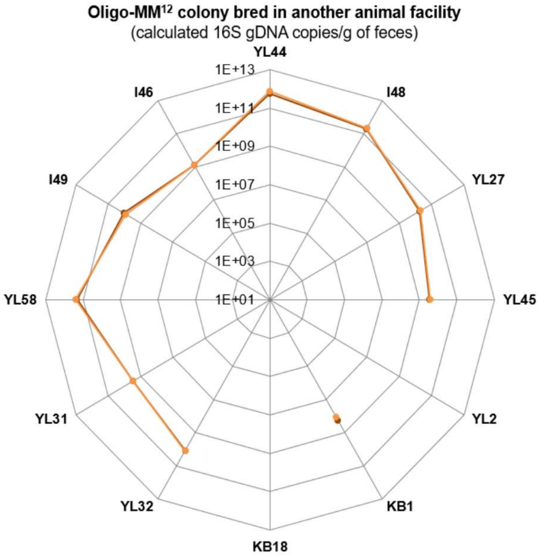

Akkermansia muciniphila YL44
Bacteroides caecimuris I48
Muribaculum intestinale YL27
Turicimonas muris YL45
Bifidobacterium animalis YL2
Enterococcus faecalis KB1
Acutalibacter muris KB18
Enterocloster clostridioformis YL32
Flavonifractor plautii YL31
Blautia coccoides YL58
Limosilactobacillus reuteri I49
Clostridium innocuum I46

Supplementary Fig. 9. Assessment of Oligo-MM^{12} gut microbiota stability in facility 2. Radar plot showing the Oligo-MM^{12} strains distribution in feces of Oligo-MM^{12} breeders (n=9) and their litters (n=17) used in this study. *Bifidobacterium animalis* YL2 and *Acutalibacter muris* KB18 strains were below qPCR detection limit. Source data are provided as a Source Data file.

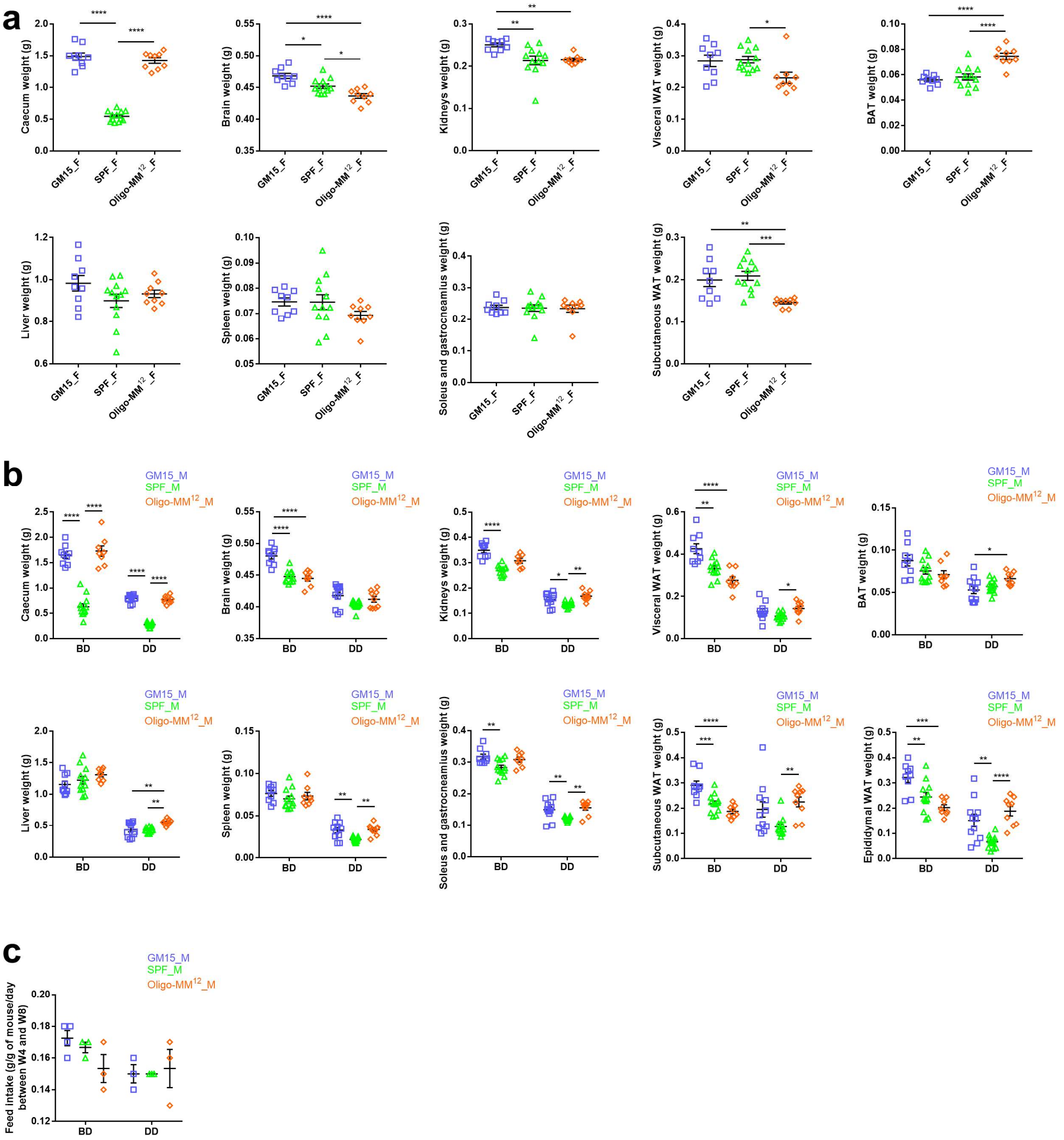

Supplementary Fig. 10. Measurements of organs weight of animals from facility 2. a and **b** Organs weight. Dot plots where dots, lines and error bars represent respectively individual mice, means and SEM. Female (F) and male (M), breeding diet (BD) and depleted diet (DD), white adipose tissue (WAT) and brown adipose tissue (BAT) respectively. GM15 mice (9 F, 9 M_BD, 11 M_DD), SPF mice (12 F, 12 M_BD, 13 M_DD except 12 M_DD for kidneys) and Oligo-MM^{12} mice (9 F, 8 M_BD except 7 M_BD for kidneys, 9 M_DD). One-way ANOVA followed by Dunn's multiple comparison analyses (visceral WAT, kidneys and soleus/gastrocneamius of females; spleen and kidneys of males under BD; brain and

subcutaneous WAT of males under DD) or Tukey's multiple comparison analyses otherwise. **c** Feed intake normalized per g of mouse per day over 4 weeks. Dot plot where dots, lines and error bars represent respectively individual cages, means and SEM. One-way ANOVA followed by Dunn's multiple comparison analysis (9 GM15_M_BD, 11 GM15_M_DD, 12 SPF_M_BD, 13 SPF_M_DD, 8 Oligo-MM^{12}_M_BD and 9 Oligo-MM^{12}_M_DD housed in 4, 3, 3, 3, 3 and 3 cages respectively). *P<0.05, **P<0.01, ***P<0.001, ****P<0.0001. Source data are provided as a Source Data file.

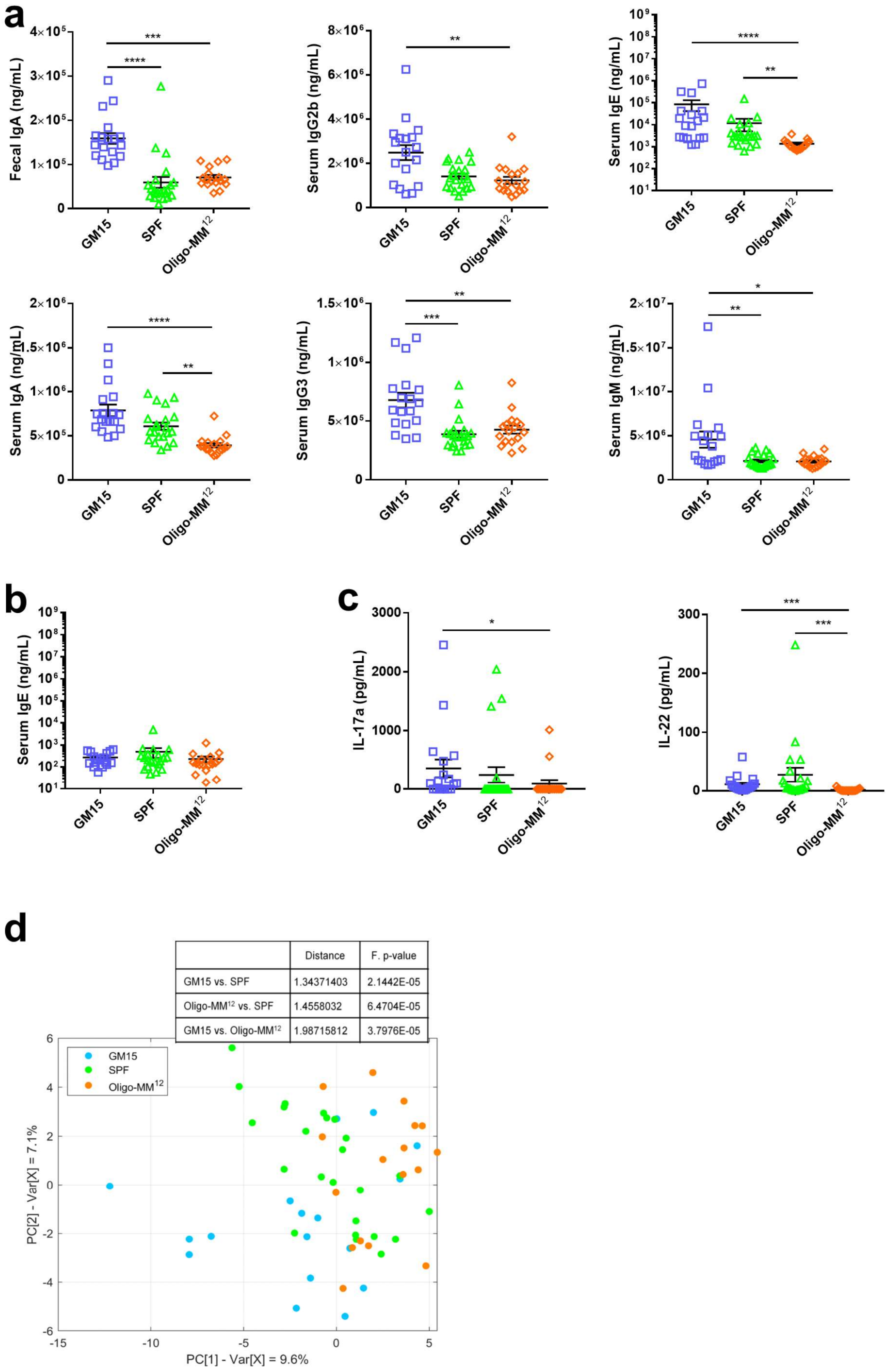

	Distance	F. p-value
GM15 vs. SPF	1.34371403	2.1442E-05
Oligo-MM12 vs. SPF	1.4558032	6.4704E-05
GM15 vs. Oligo-MM12	1.98715812	3.7976E-05

Supplementary Fig. 11. Immune phenotype and metabolic profile of animals from facility 2. a-c Dot plots where dots, lines and error bars represent respectively individual mice, means and SEM. **a** Fecal IgA, serum IgA, IgG1, IgG2a and b, IgG3, IgE and IgM Luminex analysis. One-way ANOVA followed by Dunn's multiple comparison analyses (18 GM15, 21 SPF except 22 SPF for fecal IgA and IgE, and 17 Oligo-MM^{12}). **b** IgE ELISA assay. One-way ANOVA followed by Dunn's multiple comparison analyses (17 GM15, 20 SPF and 17 Oligo-MM^{12}). **c**

Circulating IL-17a and IL-22 levels Luminex analysis. One-way ANOVA followed by Dunn's multiple comparison analyses (18 GM15, 21 SPF and 17 Oligo-MM[12]). **d** PCA score plot representing the distribution of the polar metabolite composition along the two first principal components (17 GM15, 24 SPF and 17 Oligo-MM[12]). *P<0.05, **P<0.01, ***P<0.001, ****P<0.0001. Source data are provided as a Source Data file.

Target	Phylum	Family	Forward primer sequence (5'→3')	Reverse primer sequence (5'→3')	Detection limit (CFU/g)
Bacteroides acidifaciens MD185	Bacteroidetes	Bacteroidaceae	CGTCTATCTTGCGGAAAGCA	CCTACAACTGGGGAGTTACCA	3,14E+05
Bacteroides caecimuris MD237	Bacteroidetes	Bacteroidaceae	GCAATGGGCTTTCGGTCAA	CCGAAAAGGCAGGGTTTCAA	1,77E+04
Parabacteroides goldsteinii MD072	Bacteroidetes	Tannerellaceae	CGTCACGGCCTACCAGATAA	CCTAACCGTCCACGACAAAGTA	1,63E+05
Clostridium cocleatum I50	Firmicutes	Erysipelotrichaceae	ACAGAGAGGCGAAGAAGGAC	GACGGATCTCTGCATGCTTAC	4,05E+03
Enterocloster clostridioformis YL32	Firmicutes	Lachnospiraceae	ATTGACGCCGGTATCCACAA	AGTTGCGGCAATAGGCGATA	2,45E+05
Clostridium sp. MD294	Firmicutes	Lachnospiraceae	TTGTCCTGTCCTTGCGTTCC	GCTTGAAAACAGGCGCAGAC	2,73E+02
Clostridium sp. MD300	Firmicutes	Lachnospiraceae	AAACGGGCAAATTCCAATCCC	ACTGGGCTGCTCTTTTGATACA	1,25E+04
Subtilibacillum caecimuris MD335	Firmicutes	Lachnospiraceae	GAGCTGCCTTCAAACCGTAC	GCAGACTACAGACCGACTCTTA	1,38E+04
Longibacillum caecimuris MD329	Firmicutes	Lachnospiraceae	CGTGCGCGTCAAAGGTGATA	ATCACTTGATGCGGCGCTTA	6,57E+05
Irregularicoccus caecimuris MD308	Firmicutes	Lachnospiraceae	TCCCTGATCCACACTCTTTCC	GTTGCTGCTTGACACGGTAA	5,54E+05
Lactobacillus johnsonii MD006	Firmicutes	Lactobacillaceae	TCCGCGATGTCGAGCTTAA	ACGTCAACTACGTCACCTTCA	5,90E+04
Ligilactobacillus murinus MD040	Firmicutes	Lactobacillaceae	GCCACCACGATTGATGATACC	TGGCTACAAGGGTCTTGTTGAA	9,22E+02
Limosilactobacillus reuteri MD207	Firmicutes	Lactobacillaceae	TGCTGCCGGCCTTTTATACA	CTCCGTCCATCGCGAAACTA	8,08E+02
Anaerotruncus colihominis JM4-15	Firmicutes	Ruminococcaceae	GAGGTGGGAAGTTGCCAAAA	CGTGGCTGGCTTTGAAGAA	1,63E+04
Escherichia coli Mt1B1	Proteobacteria	Enterobacteriaceae	GGCACCATTTTGCCTAGTTCC	ATCGTACCCCAAACCGACAC	2,42E+05
16S rRNA from most bacteria	NA	NA	GTGSTGCAYGGYTGTCGTCA	ACGTCRTCCMCACCTTCCTC	5,12E+05

Supplementary Table 1. GM15 strain-specific primers. Sequences of primers designed in this study and detection limits by qPCR microfluidic assay.

GM15 consortium	Facility1_GM15 (n=29) vs. Facility2_GM15 (n=27) F0 and F1 filial generations	Facility1_SOPF (n=19) vs. Facility2_SPF (n=33) 2 consecutive generations
Bacteroides acidifaciens MD185	ns	ns
Bacteroides caecimuris MD237	**	****
Parabacteroides goldsteinii MD072 #	**	no test (only 2 positive Facility2_SPF samples)
Clostridium cocleatum I50 * #	ns	**
Enterocloster clostridioformis YL32 #	ns	**
Clostridium sp. MD294 * #	no test (only 2 positive Facility1_GM15 samples)	ns
Clostridium sp. MD300 * #	ns (5 positive Facility2_GM15 samples)	ns
Subtilibacillum caecimuris MD335 ** ##	ns	****
Longibacillum caecimuris MD329 ** ##	ns	****
Irregularicoccus caecimuris MD308 ** #	ns	ns
Lactobacillus johnsonii MD006 #	ns	ns
Ligilactobacillus murinus MD040	ns	****
Limosilactobacillus reuteri MD207 #	**	no test (only 2 positive Facility2_SPF samples)
Anaerotruncus colihominis JM4-15 * #	ns (3 positive Facility1_GM15 samples)	ns
Escherichia coli Mt1B1	*	*

Supplementary Table 2. Assessment of gut microbiota reproducibility in GM15 and SOPF/SPF mice in 2 animal facilities. One-way ANOVA. Summary table of results from Dunn's multiple comparison test applied to GM15 strains concentration quantified by microfluidic qPCR assay in GM15 mice and SOPF/SPF mice between facility 1 and facility 2. Strains MD072#, I50*#, YL32#, MD294*#, MD300*#, MD308# MD006#, MD207# and JM4-15*# were at the detection limit of the qPCR microfluidic assay, and thus were not detected in all GM15 (*) or SPF (#) samples respectively. Strains MD335**##, MD329**## and MD308** were below detection limit of the qPCR microfluidic assay, and thus were not detected in any GM15 (**) or SPF (##) samples respectively. Strain YL32, obtained from the DSMZ collection, was not detected in our SOPF colony (facility 1). *P<0.05, **P<0.01, ***P<0.001, ****P<0.0001.

Target	Forward primer sequence (5'→3')	Reverse primer sequence (5'→3')	PrimeTime Eco Probe - 5' 6-FAM / ZEN / 3' IAbFQ
Clostridium innocuum I46	CGGATCGTAAAGCTCT GTTGTAAG	GCTACCGTCACTCCC ATAGCA	AAGAACGGCTCATAGAGG
Bacteroides caecimuris I48	GGCAGCATGGGAGTTT GCT	TTATCGGCAGGTTGG ATACGT	CAAACTTCCGATGGCGAC
Lactobacillus reuteri I49	GCACTGGCTCAACTGA TTGATG	CCGCCACTCACTGGT GATC	CTTGCACCTGATTGACGA
Enterococcus faecalis KB1	CTTCTTTCCTCCCGAG TGCTT	CCCCTCTGATGGGTA GGTTACC	CACTCAATTGGAAAGAGGAG
Acutalibacter muris KB18	TGGCAAGTCAGTAGTG AAATCCA	TCACTCAAGCTCGAC AGTTTCAA	CTTAACCCATGAACTGC
Bifidobacterium animalis YL2	GGGTGAGTAATGCGTG ACCAA	CGGAGCATCCGGTAT TACCA	CGGAATAGCTCCTGGAAA
Muribaculum intestinale YL27	TCAAGTCAGCGGTAAA AATTCG	CCCACTCAAGAACAT CAGTTTCAA	CAACCCCGTCGTGCC
Flavonifractor plautii YL31	AGGCGGGATTGCAAGT CA	CCAGCACTCAAGAAC TACAGTTTCA	CAACCTCCAGCCTGC
Enterocloster clostridioformis YL32	AATACCGCATAAGCGC ACAGT	CCATCTCACACCACC AAAGTTTT	CGCATGGCAGTGTGT
Akkermansia muciniphila YL44	CGGGATAGCCCTGGG AAA	GCGCATTGCTGCTTT AATCTTT	TGGGATTAATACCGCATAGTA
Turicimonas muris YL45	AGACGGCCTTCGGGTT GTA	CGTCATCGTCTATCG GTATTATCAA	ACCACTTTTGTAGAGAACGA
Blautia coccoides YL58	GAAGAGCAAGTCTGAT GTGAAAGG	CGGCACTCTAGAAAA ACAGTTTCC	TAACCCCAGGACTGCAT

Supplementary Table 3. Oligo-MM^{12} strain-specific primers and probes. Sequences of primers and probes designed by Brugiroux et al. [2].

Supplementary References

1. Yutin, N. & Galperin, M. Y. A genomic update on clostridial phylogeny: Gram-negative spore formers and other misplaced clostridia. *Environ. Microbiol.* **15**, 2631-2641 (2013).
2. Brugiroux, S., Beutler, M., Pfann, C., Garzetti, D., Ruscheweyh, H. J., Ring, D., Diehl, M., Herp, S., Lötscher, Y., Hussain, S., Bunk, B., Pukall, R., Huson, D. H., Münch, P. C., McHardy, A. C., McCoy, K. D., Macpherson, A. J., Loy, A., Clavel, T., Berry, D. & Stecher, B. Genome-guided design of a defined mouse microbiota that confers colonization resistance against Salmonella enterica serovar Typhimurium. *Nature Microbiology* **2**, 16215 (2016).
