# Supplementary material for: A standardized gnotobiotic mouse model harboring a minimal 15-member mouse gut microbiota recapitulates SOPF/SPF phenotypes
Source: Nat Commun. 2021 Nov 18;12:6686. doi: 10.1038/s41467-021-26963-9 (PMC8602333; doi:10.1038/s41467-021-26963-9)
Supplement: Supplementary file 3 — Description of Additional Supplementary Files [file 41467_2021_26963_MOESM3_ESM.docx]

**Description of Additional Supplementary Files**

**File Name:** Supplementary Data 1

**Description:** KEGG clusters. Extended list of KEGG modules and clusters from metagenomes from GM15, SOPF, Oligo-MM12 and ASF mouse models. (XLSX 135 kb).

**File Name:** Supplementary Data 2

**Description:** Polar metabolites and lipids concentration in plasma samples. The metabolites quantification was performed with the help of Chenomx NMR suite 8.6. The non-polar database profiles were created with the help of the Compound Builder module using 1H NMR spectra of authentic lipid standards. Two-tailed t-test with unpaired samples assuming unequal variances. (XLSX 192 kb).
